# Elements of Pharmacology

**Published:** 1888-09

**Authors:** 


*Elements of Pharmacology*. By Dr. OSWALD SCHMIEDEBERG, Professor of Pharmacology, University of Strasburg. Translated, under the Author's supervision, by THOMAS DIXSON, M.B., Lecturer on Materia Medica in the University of Sydney, N.S.W. Edinburgh: YOUNG J. PENTLAND. 1887.

PROFESSOR SCHMIEDEBERG'S pharmacological commentary on the last edition of the German Pharmacopœia is a work which is, to some extent, worthy of the reputation of the distinguished author. Although nominally treating of pharmacology only, it alludes also more than incidentally to the therapeutical uses of the substances treated of, and is therefore interesting to the physician as well as useful to the physiologist. The book is not one which is to be ranked in comparison with Dr. Lauder Brunton's text-book, being on a much less extended scale and less complete plan, and seems indeed brief, even to baldness, on certain substances which are useful therapeutically, though of limited sphere of action physiologically. To those, however, who do not possess Dr. Brunton's work, it can be unhesitatingly recommended.

Dr. Dixson's translation is expressed in good idiomatic English, bearing but few traces of the Teutonic phraseology of the original, though here and there we meet with a strange word, such as "feringrea," which conveys no definite meaning to most practitioners. At the time of translation, also, opportunity has been taken to incorporate some new diagrams and fresh matter, bringing the book more nearly up to date.

